# Molecular Identification and Karyological Analysis of a Rampant Aspen* Populus tremula* L. (Salicaceae) Clone

**DOI:** 10.1155/2017/5636314

**Published:** 2017-04-23

**Authors:** Dmitry V. Politov, Yuri S. Belokon, Anna V. Shatokhina, Maryana M. Belokon, Nail A. Khanov, Elena A. Mudrik, Tatyana A. Polyakova, Anna B. Azarova, Konstantin A. Shestibratov

**Affiliations:** ^1^Laboratory of Population Genetics, Vavilov Institute of General Genetics, Russian Academy of Sciences, Moscow 119991, Russia; ^2^Laboratory of Biotechnologies, Sabinsky Training Experimental Forestry, Leskhoz, Republic of Tatarstan 422062, Russia; ^3^Forest Genetics Department, Russian Center for Forest Health, Pushkino 141207, Russia; ^4^Forest Biotechnology Group, Shemyakin & Ovchinnikov Institute of Bioorganic Chemistry, Russian Academy of Sciences, Pushchino Branch, Pushchino 142290, Russia

## Abstract

A rampant highly heterozygous aspen (*Populus tremula* L.) clone* “Meshabash”* has been revealed in course of population genetic diversity analysis in a native stand in the Republic of Tatarstan, Russia. Here we report the results of karyological analysis showing that this highly vigorous clone is diploid (2*n* = 38) while typically triploid aspen demonstrates increased growth rate and resistance to aspen trunk rot caused by fungus* Phellinus tremulae*. By means of DNA identification of a series of model trees using 14 SSR loci we outlined the area occupied by this clone (at least 1.94 ha) and demonstrated that its ramets constitute 40 out of 48 genotyped trunks on the plot with the maximal distance between ramets 254 m. Since aspen is able to regenerate after cutting or die-off of maternal tree by root suckers at a distance up to 20–35 m this assumed that current stand appeared as a result of such spreading from an ortet tree during at least 5 generations. Trunk rot damage in the wood of model trees indicated low influence of this pathogen on viability and performance of the studied clone that can be associated with its extreme heterozygosity level (0.926) exceeding all the studied trees in this research plot and in three other control samples.

## 1. Introduction

Clonality is widespread in many plant groups including some forest trees of great ecological and economic value. Quaking aspen,* Populus tremula* L., as well as many other poplar species, demonstrates a mixed genetic structure when a natural stand represents a combination of trees derived from seeds able to spread by wind for long distances and root suckers responsible for fast regeneration of the best genotypes within a stand, usually after logging or fire [1]. Root suckers have high growth energy and suppress regeneration of sexually reproduced aspen and other species that leads to appearance of pure monodominant forest stand [2]. The question why particular genotypes become rampant and demonstrate high performance and sustainability compared to other conspecific individuals and representatives of other concurrent species is important for understanding formation and functioning of clonal species populations and for selection of perspective clones for using in intensive plantation forestry. Typically, aspen clones able to spread over large territories are triploid and their karyotype is usually referred to as responsible for high vigor and resistance to aspen trunk rot caused by pathogenic polypore fungus* Phellinus tremulae* (Bondartsev) Bondartsev & P.N. Borisov [3]. However, this phenomenon and fine genetic structure of mixed clonal and seed-derived populations are better studied in related North American species, trembling aspen,* Populus tremuloides* Michx. [4, 5], while for Eurasian quaking aspen data are scarce [6] and almost absent for eastern part of its range.

In this paper, we report the results of karyological analysis of a rampant quaking aspen clone* “Meshabash”* that we previously revealed in Sabinsky Experimental Forestry in the Republic of Tatarstan, Russia [7], and outline territory occupied by its ramets identified by DNA markers. We also present first pilot data on the growth rate and damage caused by aspen trunk rot in representative ramets of this clone.

## 2. Materials and Methods

### 2.1. Research Plot

The research plot was established in the Meshabash Forestry District, Sabinsky Experimental Forestry (Republic of Tatarstan, Russia), within the square 217 and Forest Management Unit 27 (FMU#27). The study area represented an almost pure aspen stand, according to forest inventory data, FMU 27 consists of 6 units of 1st-storey aspen (forest tree class 1A): 2 units of 2nd-storey aspen: 2 units of small-leaved lime* Tilia cordata* Mill.: 1 unit of birch* Betula pendula* Roth. Degree of closeness is 0.6. Undergrowth is formed by lime and red raspberry* Rubus idaeus* L. This stand is surrounded by a mixed forest formed by birch* Betula pendula* Roth. and Norway spruce,* Picea abies* (L.) H. Karst. Aspen is absent in adjacent Forest Management Units except for the northern part where sparsely scattered trees are found within 100 m from the border of FMU#27. The total area occupied by aspen and designated as FMU#27 is 2.2 ha.

### 2.2. Plant Material

For genetic studies we sampled 48 trees for which GPS-coordinates were scored. Out of these spotted trees, first 24 trees were outlining the outer border of aspen stand within Forest Management Unit 27 (FMU#27). Next 24 trees were sampled after genotyping of the first 24 trees and located inside the contour delimiting distribution of the identified ramets of the main clone. Altogether, we counted 3083 trees of the 1st and 2nd storeys (conditionally taking only trees which are thicker than 8 cm), so, out of these, the sampled 48 trees were representing 1.6% out of 3083 trunks. Among the spotted trees, 28 can be attributed to the 1st storey which represents about 11.8% out of 236 trees of the 1st storey counted on the research plot.

Since in adult aspen the crown is lifted not less than 10–15 m above the ground surface, it is extremely difficult to collect shoots with leaves and buds that are normally used for DNA extraction. Instead, for trees of the first storey, we decided to collect cambium; for that, we cut off a piece of wood with bark by axe from each model tree. Collected specimens were placed into plastic bags and stored at −70°С until DNA extraction.

In the analysis of genetic relatedness between genotypes we used genotypic data for three native stands (“Prisady” in Moscow region, “Voronezh” in Voronezh region, and “Yoshkar-Ola” in Republic of Mari-El) taken from Politov et al. [7, 8].

For karyological analysis, we used young leaves of one clone from native stand (*“Meshabash”* clone) and, as a control, roots and young leaves of ramets of three elite clones obtained from Forest Biotechnology Lab collection of the Institute of Bioorganic Chemistry of the Russian Academy of Sciences (Pushchino, Russia):* PtV22, Line#4*,* Understory-3*. Each specimen was analyzed in two or three replicates.

### 2.3. DNA Extraction

We extracted DNA from the scraped off surface of wood in the zone of its contact with bark. Tissue represented a cambium layer and part of surrounding wood. About 750–1000 mg of these tissues was homogenized and used for DNA isolation by a modified cethyltrimethylammonium bromide (CTAB) protocol [9, 10].

### 2.4. Microsatellite Analysis

The previously selected set of 14 microsatellite loci* ORPM193*,* ORPM202*,* ORPM206*,* ORPM220*,* ORPM296* [11],* WPMS14*,* WPMS15*,* WPMS16*,* WPMS17*,* WPMS18*,* WPMS19*,* WPMS20*,* WPMS21*, and* WPMS22* [12] was employed for DNA identification [7, 8] of “*Meshabash*” clone ramets from putatively growing together in the same plot other aspen genotypes/clones.

Reagents used for PCRs, laboratory equipment, regimes of DNA amplification, fragment analysis, and gel documentation were performed as described earlier [7, 8]. PCR products were analyzed by electrophoresis in vertical blocks of 6% polyacrylamide gel in the tris-EDTA-borate buffer system. After electrophoresis, the gels were stained in a solution of ethidium bromide and visualized under UV light and their graphic images were captured using the Doc-Print II (Vilber Lourmat) gel documentation system. The size of amplified fragments was estimated with the help of the Photo-Capt software.

### 2.5. Statistical Analysis

Clone identity was determined using a function “multilocus matches analysis for codominant data” in the add-in for MS Excel, GenAlEx 6.5 [13, 14] (see Supplementary Materials available online at https://doi.org/10.1155/2017/5636314). Values of pairwise relatedness [15] among genotypes were calculated and averaged within stands. Check for normality of the distribution of pairwise relatedness (Figure 4) was done in* STATISTICA* (StatSoft Inc.) using Kolmogorov-Smirnov and Lilliefors test (Lilliefors) and Shapiro-Wilk's W (SW-W) tests.

### 2.6. Karyological Analysis

We used modified protocol for study of chromosomes of fruit trees based on temporary crushed preparations of parenchyma of developing roots or leaves [16]. Material was fixed by Carnoy liquid (3 parts of ethanol and 1 part of glacial acetic acid). Pretreatment was performed with cold water for 12 to 24 hours at +2°C. In case of necessity of long-time storage of the fixed material we changed the fixation solution twice with 70° ethanol. Before staining, material was placed in 4% iron alum solution for 20–30 min. Preparations were stained with 1% hematoxylin solution. For reliability and reproducibility of results, we studied karyotypes of up to 10 roots and leaves from each individual plant and in each preparation we counted chromosomes in 5 to 10 metaphase plates. Chromosome numbers were determined by using microscopes Biomed-2 (Biomed-Service Ltd., Moscow, Russia) and Axioskop-40 (Carl Zeiss AG, Oberkochen, Germany).

## 3. Results

### 3.1. DNA Identification and Analysis of Clonal Structure

**Figure 1:**
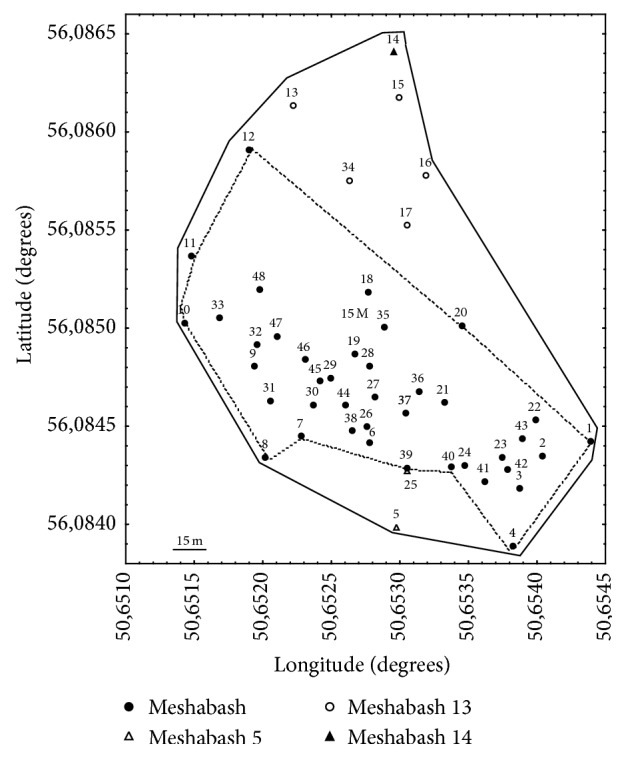
Spatial distribution of the studied trees. Different markers represent genets. A solid line delimits distribution of aspen within FMU#27; spotted line outlines positions of outermost trees belonging to* “Meshabash” *clone.

By using a set of 14 SSR (Simple Sequence Repeat or microsatellite) loci we identified genotypes of 48 trees spotted by GPS.  Figure 1 shows spatial distribution of the studied trees. Comparison of the first 24 specimens, collected on the aspen border outline, has shown that they all belong to four multilocus genotypes. Two trees (Meshabash#2 and Meshabash#25) on the southern edge of the plot represented genotype* “Meshabash#5”*; five trees in the north at the periphery were identified as separate genotype* “Meshabash#13”* and one more tree on the northern edge was also genetically different and named as* “Meshabash#14”*; all other 16 trees possessed the same genotype that was identical to the previously described as* “Meshabash”* clone [7]. Additional collection of 24 trees inside the contour formed by outermost SSR-identified ramets was done. SSR analysis of these trees (##25-48) showed that all of them belong to* “Meshabash” *clone. That initial description [8] was based on 29 unspotted trees of the second storey representing ramets of the same rampant clone. Data presented here clearly demonstrated complete identity of the studied 40 GPS-spotted trees to this earlier described clone for all 14 microsatellite loci. So altogether we identified 69 ramets of* “Meshabash”* clone and only eight trees belonging to five other genotypes, all located on the periphery of the plot.

### 3.2. Analysis of Genetic Relatedness and Heterozygosity

**Figure 2:**
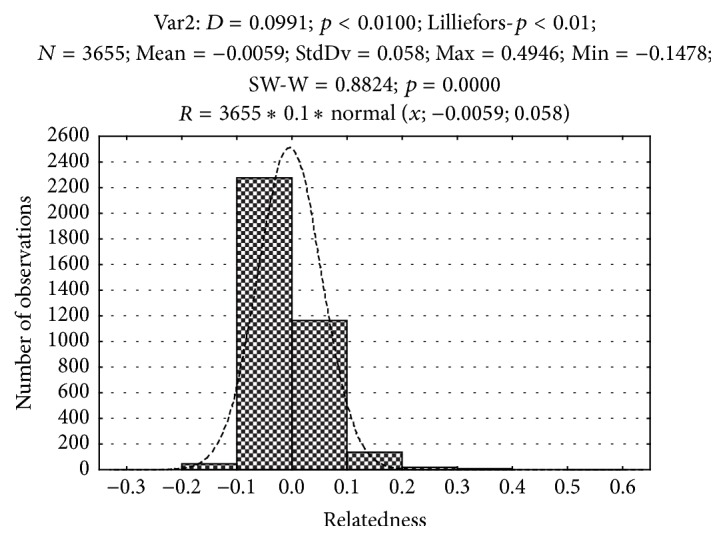
Distribution of pairwise relatedness in aspen natural stands (ramets of the same clone are excluded).

**Figure 3:**
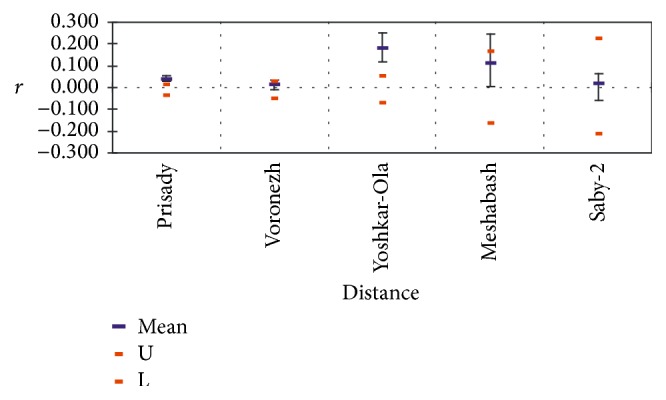
Mean within population pairwise values of relatedness.

Pairwise relatedness [15] distribution was deviated significantly from normal indicating some related genotypes. The tail in the right-hand part of the histogram shows* R* values characteristic to half sibs (about 0.25) and sibs (about 0.5); however, values close to 0.9–0.95 that would be signaling on the presence of somaclonal variation due to mutations in particular loci were not detected (Figure 2). Mean within population pairwise values of relatedness (Figure 3) were found to be near zero in predominantly seed-originated stands “Prisady” and “Voronezh” and in a small sample from Sabinsky Forestry (“Saby-2”). However, R demonstrated positive mean values on the studied plot in Tatarstan represented predominantly by* “Meshabash”* clone and in the sample from Mariy-El Republic “Yoshkar-Ola” where we also detected only 13 different genotypes among 32 studied trees [8]. In these cases, the increasing participation of rampant clones in pollination and seed production leads to the raise of relatedness among genotypes within stands because most seed descendants of the represented clones are results of crossings among relatives.

**Figure 4:**
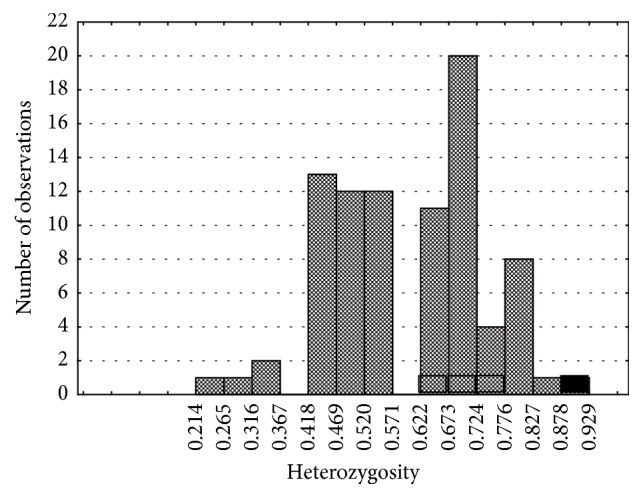
Distribution of individual heterozygosity in aspen natural stands (ramets of the same clone are excluded). Black box:* “Meshabash” *clone; open boxes: other clones found within the research plot (FMU#27).

We have already reported on the highest individual heterozygosity value (13 out of 14 microsatellite loci) of* “Meshabash”* clone among all genotypes studied in several natural populations [7]. Here we present these data on the distribution of individual heterozygosity supplemented by genotypes #5, #13, and #14 revealed on the research plot during current more detailed study of clonal structure of the stand. These three additional genotypes were heterozygous for 9, 10, and 8 microsatellite loci, respectively (Figure 4).

### 3.3. Karyology

**Figure 5:**
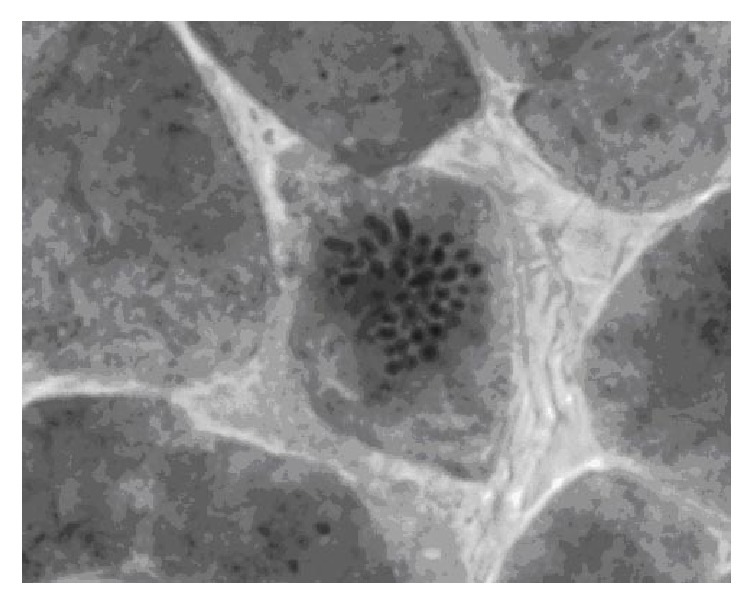
Metaphase plate of aspen clone* Meshabash*: 2*n* = 38.

The karyological analysis has shown that the rampant clone* “Meshabash”* was diploid with chromosome number typical for quaking aspen 2*n* = 38 (Figure 5).

**Figure 6:**
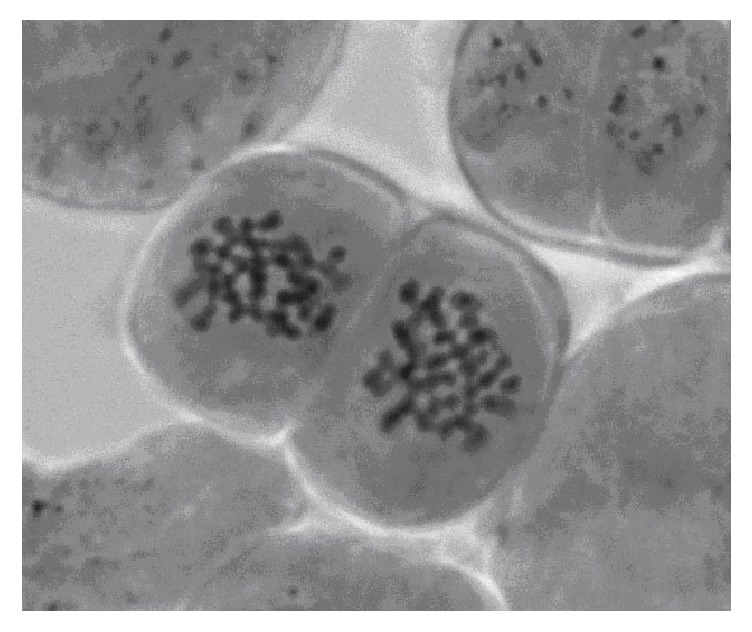
Metaphase plate of aspen clone* PtV-22*: 2*n* = 38.

**Figure 7:**
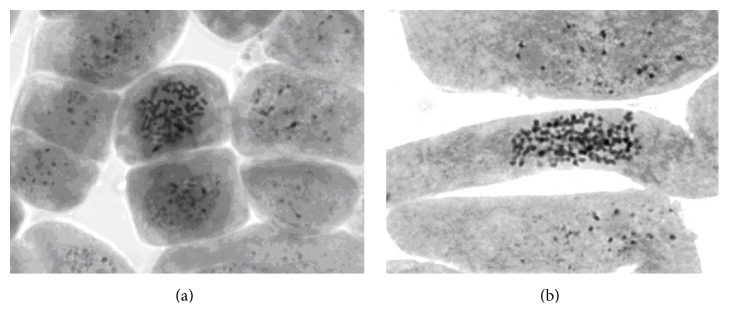
Metaphase plate of aspen clone* Line#4*: (a) 2*n* = 38 and (b) 2*n* = 3*x* = 57.

Among the studied control specimens of elite clones, one clone (*PtV22*) was also diploid (Figure 6) while clones* Line#4* and* Understory-3* were shown to be mixoploid (Figure 7), represented by diploid 2*n* = 38 and triploid (2*n* = 3*x* = 57) cells found in different proportions (Table 1).

### 3.4. Wood Quality and Trunk Rot Damage

**Figure 8:**
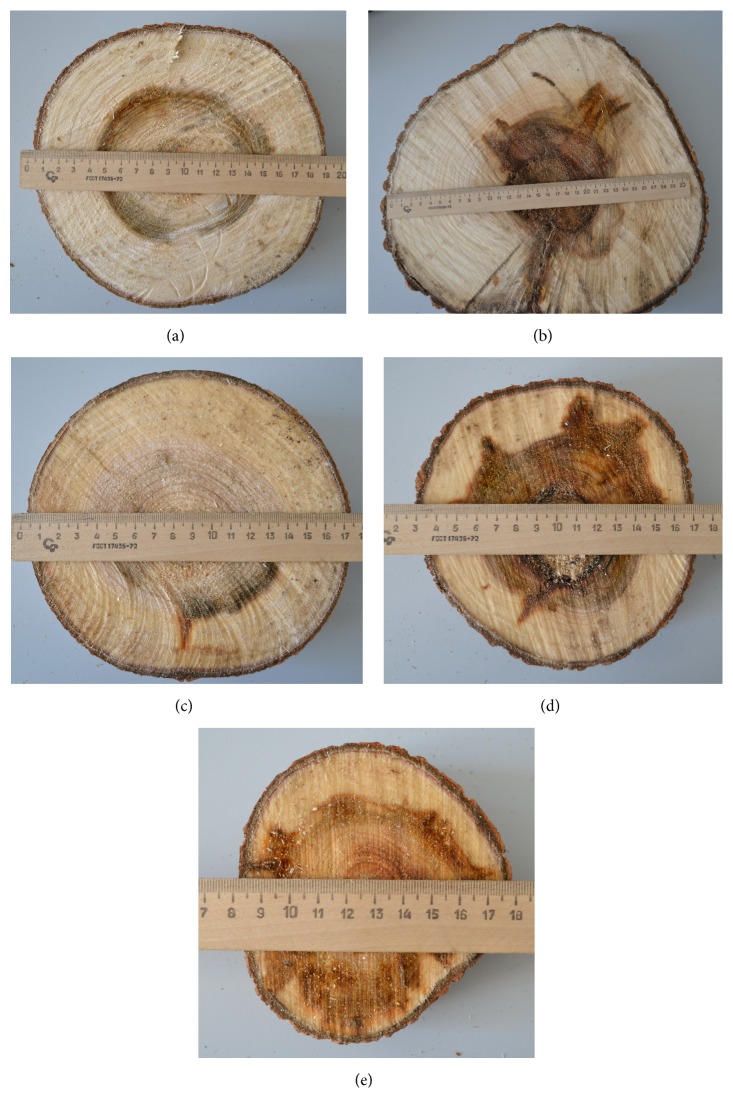
Transversal saw-cuts of model trees of* “Meshabash”* clone for evaluation of age, growth rate, and damage by aspen trunk rot ((a) #6; (b) #7; (c) #8; (d) #101; (e) #102).

**Figure 9:**
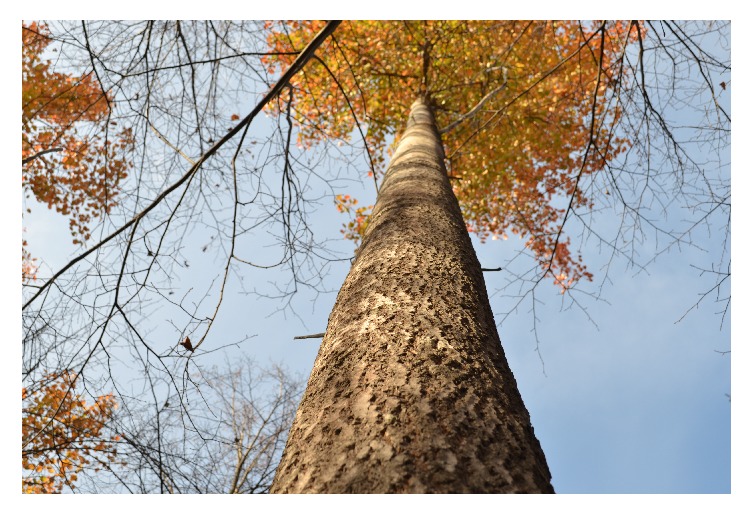
A typical 1st-storey aspen tree of* “Meshabash”* clone demonstrating high vigor and growth.

**Figure 10:**
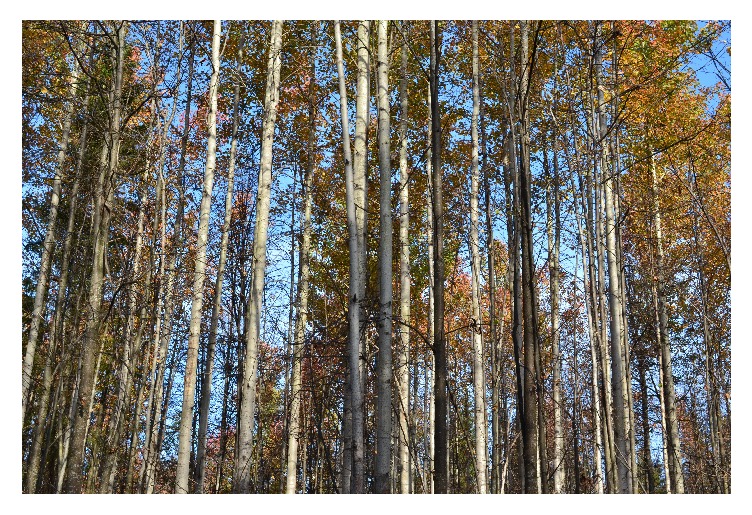
Dense aspen forest that originated from root suckers (ramets) of* “Meshabash”* clone.

Five model trees, three out of the numbered and spotted trees of the first storey (Meshabash#6, Meshabash#7, and Meshabash#8) and two unspotted of the second storey (denominated as Meshabash#101, Meshabash#102), were cut off in order to evaluate their growth rate and degree of aspen trunk rot damage to wood (Figure 8, Table 2). Growth rate corresponded to the highest growth rate classes, 1a or 1b [17]. Damage from* Phellinus tremulae *was however significant, and two trees of the 2nd storey and one tree of the 1st storey have been strongly or moderately damaged (Figure 8). However, presence of aspen trunk rot did not cause slowing down of growth and development of trees that originated from root suckers (Figures 9 and 10).

## 4. Discussion

Aspen is characterized by mixed regeneration mode including both seed reproduction and vegetative regeneration by root suckers [1, 17]. Despite the fact that aspen tree may produce millions of seeds, sexual reproduction is hampered by low pollination level, low viability and weight of seeds, and their high susceptibility to environmental conditions (especially drought) during germination [18, 19]. These factors lead to the situation when aspen seedlings are outcompeted by grass, other tree species, and root suckers of own species. All this explains why seed regeneration is relatively rare in natural aspen stands. Seed reproduction is most important at large scale of distances while closely located trunks often represent ramets derived from the same ortet. In aspen, roots may survive long even after death of maternal tree and trunk sprouts appear from dormant buds and then develop into new trunks [1].

For North American trembling aspen, relationships between clone size and age (estimated as a rate of somaclonal mutations in a pool of ramets by 14 SSR loci) were studied [20]. The authors found poor correlation between these parameters and concluded that clone size is not a good proxy for its age. Analysis of clonality in* Populus tremula* in Northern Europe revealed quite small clone size, 2.3 ramets per clone with 70% of clones represented by just one ramet [6]. In the case of the studied here quaking aspen clone* “Meshabash,”* the area occupied was at least 1.94 ha with the maximal detected distance between trees 254 m. Since aspen is able to regenerate after cutting or die-off of maternal tree by root suckers at a distance up to 20–25 m with a recorded maximum at 33.5 m [21] this implied that current stand appeared as a result of such spreading from a founder (ortet) tree putatively located in the center of plot during at least 5 cycles of dieback and regeneration.

In a natural population of woody plant, polyploidy and in particular its most usual case, triploidy, may result from fertilization of normal haploid ovule with unreduced pollen or fertilization of unreduced ovule with normal pollen. Triploids naturally occurring in a population may be sterile or have reduced fertility but are able to persist for ages as vegetative clones. There are numerous evidences for the adaptive advantages of such clones [3]. Most prominent feature of triploid aspen is its resistance to trunk rot. In our case, the widespread clone was shown to be diploid with chromosome number typical for quaking aspen, 2*n* = 38 [22].

Giant quaking aspen clones with exclusive growth [23] have been described in several provinces of Sweden in 1930s and were shown to be triploid [24, 25]. These trees were characterized by thicker twigs and larger leaves and buds and trunk sections exhibited high resistance of triploid giant trees to trunk rot as compared to normal diploid aspen from the same stand [24].

Triploidy is widespread in trembling aspen* Populus tremuloides *across North America [5], and its frequency and the size of persisting triploid clones decrease from southwest to northeast. The authors associate triploidy advantage with unglaciated drought-prone regions where ecological factors favor clonality.

*Populus nigra* clonality occurs in several forms [26]: (1) ramets originated from root suckers and representing small compact groups and (2) distant, separated by up to 19 km, identical (by five SSR loci) genotypes appeared evidently due to downstream dispersal of vegetative tree fragments by Garonne River. Comparison among young, middle-aged, and old stands showed that proportion of clonal individuals was the lowest in young stands and genetic diversity was maximal in middle-aged stands.

Besides triploidy, heterosis can also explain the high vigor of selected clones. Most data on this effect refer to interspecific hybrids that often demonstrate faster growth and increased resistance as compared to interspecific crosses [27, 28]. High heterozygosity of successful rampant clones may indicate the effect of overdominance, and the studied* “Meshabash”* clone is a good example of heterozygote superiority. Despite the fact that microsatellite loci are generally selectively neutral (except for the cases when they are closely genetically linked to functionally loaded genes) high heterozygosity for them may indicate overall high genome heterozygosity.

## 5. Conclusion

The presented results based on efficient molecular identification of individual* Populus tremula* genotypes demonstrate that a rampant aspen clone can occupy vast territory. We show that the revealed “superclone” was diploid and only moderately resistant to aspen trunk rot. Its high vitality even in the presence of the pathogen can be explained by its extremely high genome heterozygosity that we suppose is based on comparative microsatellite data. Adaptive advantage of heterozygous clones can be based on their more flexible biochemical and physiological reactions on the environmental challenges and competition with other species and other aspen clones, which originated from both seeds and root suckers. This effect and distribution of various clones in forest stands depending on the level of their heterozygosity deserve further attention and research efforts.

## Supplementary Material

Table S1: Multilocus genotypes and individual heterozygosities for 14 microsatellite loci of genets revealed during analysis of genetic diversity in four natural populations of *Populustremula*.
Table S2: Martix of Queller and Goodnight (1989) pairwise relatedness among aspen genotypes.
Table S3: Summary of mean within population pairwise relatedness (Queller and Goodnight 1989) values.

## Figures and Tables

**Table 1:** Chromosome numbers in cells of the studied aspen clones.

Specimen	Number of screened cells	Cells (number/%)	
		2*n* = 2*x* = 38	2*n* = 3*x* = 57
*“Meshabash”*	28	28/100	—
*“Ptv-22”*	62	62/100	—
*“Line#4”*	79	65/82.3	14/17.7
*“Understory-3”*	32	12/37.5	20/62.5

**Table 2:** Tree height, diameter, age, and degree of damage by aspen trunk rot of model trees of *“Meshabash”* clone.

Tree number	Diameter, mm	Height, m	Age	Trunk rot damage
#6	165	18	26	Weak
#7	330	22	cf. 30	Medium
#8	185	19	28	Weak
#101	140	17	20	Strong
#102	100	14.5	24	Strong
